# Pioglitazone and Deoxyribonucleoside Combination Treatment Increases Mitochondrial Respiratory Capacity in m.3243A>G MELAS Cybrid Cells

**DOI:** 10.3390/ijms21062139

**Published:** 2020-03-20

**Authors:** Harrison J. Burgin, M. Isabel G. Lopez Sanchez, Craig M. Smith, Ian A. Trounce, Matthew McKenzie

**Affiliations:** 1 School of Life and Environmental Sciences, Faculty of Science, Engineering and Built Environment, Deakin University, Geelong 3216, Australia; hburgin@deakin.edu.au; 2 Centre for Eye Research Australia, Royal Victorian Eye and Ear Hospital, Melbourne, Victoria 3002, Australia; isabel.lopez@unimelb.edu.au (M.I.G.L.S.); i.trounce@unimelb.edu.au (I.A.T.); 3 Ophthalmology, University of Melbourne, Department of Surgery Melbourne, Victoria 3000, Australia; 4 School of Medicine, Faculty of Health, Deakin University, Geelong 3216, Australia; craig.smith@deakin.edu.au; 5 Centre for Innate Immunity and Infectious Diseases, Hudson Institute of Medical Research, Melbourne 3168, Australia; 6 Department of Molecular and Translational Science, Monash University, Melbourne 3168, Australia

**Keywords:** mitochondrial biogenesis, mitochondrial disease, oxidative phosphorylation, OXPHOS, pioglitazone, deoxyribonucleosides, MELAS, cybrid

## Abstract

The lack of effective treatments for mitochondrial disease has seen the development of new approaches, including those that aim to stimulate mitochondrial biogenesis to boost ATP generation above a critical disease threshold. Here, we examine the effects of the peroxisome proliferator-activated receptor γ (PPARγ) activator pioglitazone (PioG), in combination with deoxyribonucleosides (dNs), on mitochondrial biogenesis in cybrid cells containing >90% of the m.3243A>G mutation associated with mitochondrial encephalopathy, lactic acidosis, and stroke-like episodes (MELAS). PioG + dNs combination treatment increased mtDNA copy number and mitochondrial mass in both control (CON) and m.3243A>G (MUT) cybrids, with no adverse effects on cell proliferation. PioG + dNs also increased mtDNA-encoded transcripts in CON cybrids, but had the opposite effect in MUT cybrids, reducing the already elevated transcript levels. Steady-state levels of mature oxidative phosphorylation (OXPHOS) protein complexes were increased by PioG + dNs treatment in CON cybrids, but were unchanged in MUT cybrids. However, treatment was able to significantly increase maximal mitochondrial oxygen consumption rates and cell respiratory control ratios in both CON and MUT cybrids. Overall, these findings highlight the ability of PioG + dNs to improve mitochondrial respiratory function in cybrid cells containing the m.3243A>G MELAS mutation, as well as their potential for development into novel therapies to treat mitochondrial disease.

## 1. Introduction

Mitochondrial disease affects approximately 1 in 4300 people, and causes significant morbidity and mortality [1]. Patients commonly suffer from debilitating, multi-systemic metabolic disorders, including brain, heart, and skeletal muscle dysfunction. There is currently no cure for mitochondrial disease, with treatment commonly consisting of a “mitochondrial cocktail” that is comprised of various vitamins and cofactors. Although a standardized formulation does not exist, most cocktails include CoQ_10_, l-carnitine, creatine, α-lipoic acid, and certain B-vitamins [2]. While individual case studies suggest improved symptoms in some patients [3], few clinical trials have examined the efficacy of these compounds, with a Cochrane review of mitochondrial therapies finding no clear evidence to support the use of any of these supplements [4].

This lack of efficacy has driven the development of new therapies to treat mitochondrial disease, with a number of clinical trials completed or now in progress [5]. New drugs that are being tested for mitochondrial disease treatment include molecules with antioxidant properties, such as KH176 [6] and EPI-743 [7], metabolites designed to boost mitochondrial oxidative phosphorylation (OXPHOS) function, such as idebenone [8], riboflavin [9], dichloroacetate [10], pyruvate [11], and 5-aminolevulinic acid [12], or molecules that protect mitochondrial inner membrane phospholipids, such as MTP-131 (elamipretide/Bendavia) [13].

Another possible approach for treating mitochondrial disease is the stimulation of mitochondrial biogenesis, which aims to boost ATP generation above a critical disease threshold [14]. Mitochondrial biogenesis is controlled by the peroxisome proliferator-activated receptor (PPAR) family of genes (α, β, and γ), as well as the transcription factor PPARγ coactivator-1α (PGC-1α), which is recognized as the “master regulator” of mitochondrial biogenesis [15]. PGC-1α can induce the expression of nuclear respiratory factor 1 (NRF1) or NRF2, which subsequently binds to the promoter region of the mitochondrial transcription factor A (TFAM) to increase its expression [16]. TFAM then drives the replication and transcription of the mitochondrial genome, stimulating mitochondrial biogenesis via the increase of steady-state mitochondrial protein levels [14,17].

One molecule that stimulates mitochondrial biogenesis via the PPAR signalling pathway is the FDA-approved drug pioglitazone (PioG), an anti-hyperglycemic PPARγ agonist used to treat diabetes mellitus [18]. PioG has been shown to induce mitochondrial biogenesis in adipose tissue in Type II diabetic patients [19], with improved insulin sensitivity correlating closely with increased mitochondrial protein expression [20]. Consequently, PioG may also be effective for treating mitochondrial disease via the stimulation of mitochondrial biogenesis in affected patients. 

A common feature of enhanced mitochondrial biogenesis is a concomitant increase in mitochondrial DNA (mtDNA) copy number, such that it is a useful marker of mitochondrial biogenesis [21]. Administration of deoxyribonucleotide triphosphates (dNTPs) or deoxyribonucleosides (dNs) can increase mtDNA copy number in a range of different cell types in vitro [22]. In addition, dNs have been used therapeutically to increase mtDNA copy number in patients with mitochondrial thymidine kinase 2 deficiency, where they bypass the nucleotide generation defect that causes mtDNA depletion in these patients [23].

As such, both PioG and dNs may be suitable for treating mitochondrial disease via the increase of mtDNA copy number and stimulation of mitochondrial biogenesis. Furthermore, by combining both compounds, it may be possible to stimulate mitochondrial biogenesis via complementary signalling pathways, resulting in lower therapeutic doses that will minimise the possibility of toxic side effects, particularly if they are to be used long-term.

To test this, we have established a unique combination of PioG and dNs to stimulate mitochondrial biogenesis in 143B cybrid cells containing the mitochondrial encephalopathy, lactic acidosis, and stroke-like episodes (MELAS) m.3243A>G mutation. This is the most common mtDNA mutation associated with mitochondrial disease, with a population frequency ranging from 4:100,000 in norther European populations to 236:100,000 in Australia [24,25]. The mutation commonly causes an OXPHOS complex I deficiency in MELAS patients, with combined deficiencies in complexes I, III, and IV also identified in some patients [26,27]. Chronic progressive external ophthalmoplegia (CPEO) and maternally inherited diabetes and deafness (MIDD) are also associated with the m.3243A>G mutation, with all patients described being heteroplasmic [28]. 

We treated cybrids with >90% of the m.3243A>G mutation simultaneously with PioG and dNs (PioG + dNs). This combination was able to increase in mtDNA copy number and mitochondrial mass, with an associated rise in maximal mitochondrial respiration rates and cell respiratory control ratios to levels comparable to untreated control cybrid cells. While PioG + dNs treatment did not increase steady-state levels of mature OXPHOS holocomplexes in m.3243A>G cybrids (as was observed for control cybrids), our findings highlight the ability of PioG + dNs to increase respiratory capacity in the presence of disease-causing mutations, as well as the potential for PioG + dNs to be developed into new therapies for mitochondrial disease.

## 2. Results

### 2.1. Combination Treatment with Pioglitazone (PioG) and Deoxyribonucleosides (dNs) Increases mtDNA Copy Number and Mitochondrial Mass in Both Control and m.3243A>G Mutant Cybrids

To stimulate mitochondrial biogenesis, 143B cybrid cells containing either wild-type mtDNA (control (CON)) or the m.3243A>G mutation (MUT) were treated with either (a) 10 µM pioglitazone (PioG); (b) a combination of four deoxyribonucleosides (dNs), containing 50 µM dG, 1 µM dC, 1 µM dA, and 1 µM dT; or (c) a combination of both 10 µM PioG and dNs (PioG + dNs). These concentrations of PioG and dNs are based on previously published studies and correspond to the minimum concentrations required to significantly increase mtDNA copy number [22,29,30].

**Figure 1:**
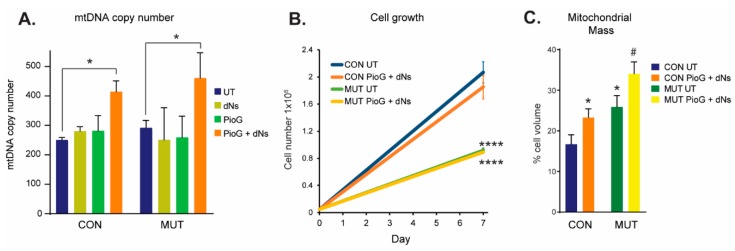
Effect of seven days of PioG + dNs combination treatment in control (CON) cybrids and m.3243A>G (MUT) cybrids. (**A**) MtDNA copy number was increased following PioG + dNs treatment in both CON and MUT cybrids, but was not affected by treatment with either PioG or dNs alone. * *p* < 0.05 (two-way ANOVA; factors: cell type and treatment, degrees of freedom (DF) = 3, F-ratio (F) = 11.46). (**B**) Cell proliferation was greater in untreated CON cybrids compared to untreated MUT cybrids; however, PioG + dNs treatment did not affect cell growth of either cell type. **** *p* < 0.0001 (two-way ANOVA; factors: cell type and treatment, DF = 1, F = 237.9). (**C**) Mitochondrial mass was significantly higher in untreated MUT cybrids compared to untreated CON cybrids, with PioG + dNs treatment increasing mitochondrial mass in both CON and MUT cybrids. * *p* < 0.05 compared to untreated (UT) CON; # *p* < 0.05 compared to UT MUT (Student’s *t*-tests). Values shown are mean ± standard deviation (s.d.).

Following treatment for seven days, mtDNA copy number was assessed as a marker of mitochondrial biogenesis in both CON and MUT cybrids (Figure 1A). Treatment with PioG alone, or with dNs alone, did not alter mtDNA copy number in either CON or MUT cybrid cells. However, simultaneous treatment with both PioG and dNs together (PioG + dNs) resulted in a 66% increase of mtDNA copy number in CON cybrids (*p* < 0.05) and a 55% increase in MUT cybrids (*p* < 0.05) compared to their respective untreated (UT) controls (Figure 1A). As individual PioG or dNs treatment did not affect mtDNA copy number (ipso facto mitochondrial biogenesis), we focused on the PioG + dNs combination treatment for subsequent experiments.

We next assessed whether PioG + dNs treatment affects cell growth and proliferation (Figure 1B). After seven days, cell numbers of untreated MUT cybrids were significantly lower than those of untreated CON cybrids (*p* < 0.0001). Notably, combination PioG + dNs treatment had no effect on the proliferation of either CON cybrids or MUT cybrids, with cell numbers the same as their respective untreated cybrids (CON, *p* = 0.303; MUT, *p* > 0.999) (Figure 1B). 

Untreated MUT cybrid cells had significantly higher mitochondrial mass (25.9% ± 17.4% of cytoplasmic volume) than CON cybrids (16.7% ± 14.3%, *p* < 0.05), as measured by confocal microscopy (Figure 1C), while treatment with PioG + dNs increased mitochondrial mass in both CON and MUT cybrids (* *p* < 0.05 compared to UT CON, and # *p* < 0.05 compared to UT MUT) (Figure 1C).

We also assessed whether mutant load was altered in MUT cybrid cells by PioG + dNs treatment. After seven days, the percentage of m.3243A>G mutation decreased slightly but significantly in the MUT cybrid cells from 99.2% ± 0.8% to 93.8% ± 2.1% (*p* < 0.05).

### 2.2. PioG + dNs Treatment Increases mtDNA Transcript Levels in Control Cybrids, but Not in MUT Cybrids 

**Figure 2:**
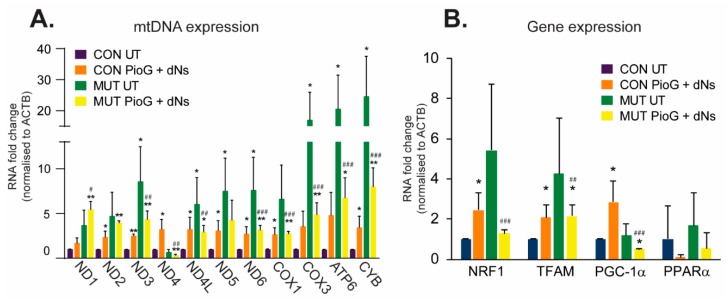
Effect of seven days of PioG + dNs combination treatment on gene expression in control (CON) cybrids and m.3243A>G (MUT) cybrids. (**A**) Expression of mtDNA-encoded transcripts was higher in untreated MUT cybrids compared to untreated CON cybrids. PioG + dNs treatment increased expression in CON cybrids, but reduced expression in MUT cybrids. (**B**) Expression of nuclear-encoded genes associated with mitochondrial biogenesis was increased in CON cybrids following PioG + dNs treatment, but was decreased in MUT cybrids. UT = untreated; values shown are mean ± s.d. * *p* < 0.05, ** *p* < 0.01, relative to untreated CON values; ^#^ *p* < 0.05, ^##^ *p* < 0.01, ^###^ *p* < 0.001 relative to untreated MUT values (Student’s *t*-tests).

After seven days of PioG + dNs treatment, CON cybrids exhibited significant increases in almost all of the mtDNA-encoded transcript levels examined, including ND2, ND3, ND4, ND4L, ND5, and ND6 (complex I), COX1 (complex IV), and CYB (complex III) (*p* < 0.05) (Figure 2A). Nuclear-encoded transcription factors associated with mitochondrial biogenesis, including NRF1, TFAM, and PGC-1α, were also significantly upregulated in CON cybrids following seven days of treatment, with PPARα unaltered (Figure 2B). 

Notably, the expression of almost all mtDNA transcripts was higher in untreated MUT cybrids compared to untreated CON cybrids (*p* < 0.05), suggesting a compensatory mechanism of elevated mtDNA gene expression in these cells to counteract the effects of the m.3243A>G mutation (Figure 2A). However, mtDNA gene expression was attenuated in MUT cybrids by PioG + dNs treatment, with almost all transcripts reduced compared to untreated MUT cybrids (Figure 2A). The only exception was ND1, which trended towards an increase in expression following PioG + dNs treatment (although this was not significant). 

While mtDNA gene expression was generally reduced in MUT cybrids following PioG + dNs treatment, transcript levels were similar to those observed in treated CON cybrids, and still significantly higher than those in untreated CON cybrid levels (* *p* < 0.05, ** *p* < 0.01) (Figure 2A). This finding suggests that PioG + dNs treatment may reduce mtDNA gene expression in MUT cybrids so that transcript levels are optimal for enhanced mitochondrial biogenesis (as they are now similar to those observed in treated CON cybrids). 

Changes in MUT cybrid nuclear gene expression following PioG + dNs treatment reflect the reduction in mtDNA gene expression, with NRF1, TFAM, and PGC-1α transcripts reduced (*p* < 0.05) (Figure 2B). However, similar to mtDNA transcripts, the expression of NRF1 and TFAM in treated MUT cybrids was reduced to similar levels as those observed in treated CON cybrids (Figure 2B). In contrast, PGC-1α expression in treated MUT cybrids was significantly lower than in untreated CON cybrids (*p* < 0.05) with PPARα expression unaltered (Figure 2B).

### 2.3. PioG + dNs Treatment Increased Oxidative Phosphorylation Protein Steady-State Levels in Control Cybrids, but Not in MUT Cybrids 

**Figure 3:**
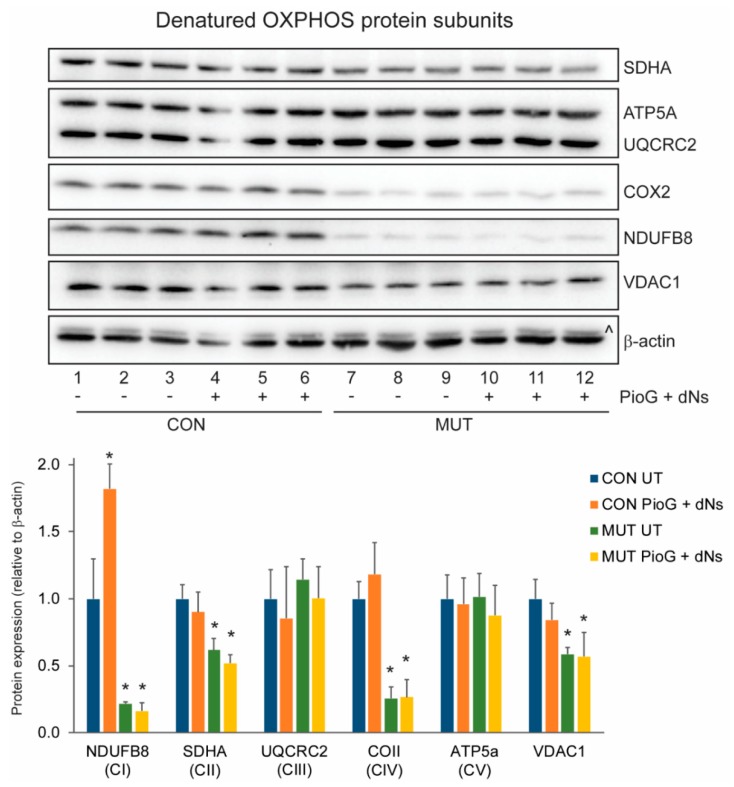
Effect of seven days PioG + dNs combination treatment on individual oxidative phosphorylation (OXPHOS) subunit steady-state levels in control (CON) cybrids and m.3243A>G (MUT) cybrids. Levels of NDUFB8, SDHA, COII, and voltage-dependent anion-selective channel protein 1 (VDAC1) were lower in untreated MUT cybrids compared to untreated CON cybrids. PioG + dNs treatment resulted in the increase in NDUFB8 in CON cybrids, but did not alter levels of any other proteins in either CON or MUT cybrids. UT = untreated; ^, UQCRC2 detected from previous immunodecoration. Values shown are mean ± s.d. * *p* < 0.05 relative to untreated CON values (Student’s *t*-tests).

**Figure 4:**
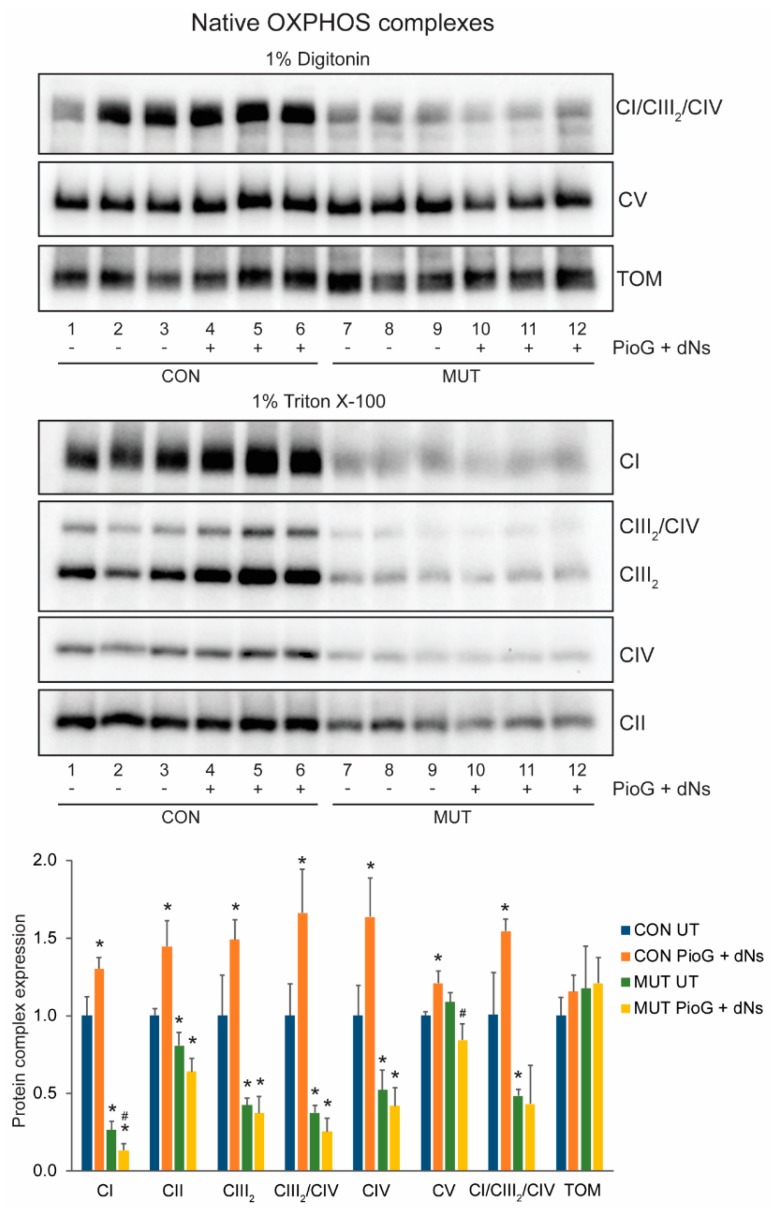
Effect of seven days of PioG + dNs combination treatment on mature OXPHOS complex steady-state levels in control (CON) cybrids and m.3243A>G (MUT) cybrids. Levels of complex I (CI), complex II (CII), the complex III dimer (CIII_2_), complex IV (CIV), the CIII_2_/CIV supercomplex, and the CI/CIII_2_/CIV supercomplex were lower in untreated MUT cybrids compared to CON cybrids. PioG + dNs treatment resulted in the increase in all OXPHOS complex and supercomplex levels in CON cybrids, but did not increase any complexes in MUT cybrids. UT = untreated. Values shown are mean ± s.d. * *p* < 0.05 relative to untreated CON values; ^#^ *p* < 0.05 relative to untreated MUT values (Student’s *t*-tests).

We next examined the effects of PioG + dNs treatment on the steady-state levels of individual OXPHOS protein subunits using SDS-PAGE (Figure 3), or the levels of mature OXPHOS complexes using blue-native polyacrylamide gel electrophoresis (BN-PAGE) (Figure 4). In untreated cells, the levels of NDUFB8 (21.6%, *p* < 0.05), SDHA (61.8%, *p* < 0.05), and COII (25.7%, *p* < 0.005) were reduced in MUT cybrids compared to CON cybrids, with similar levels of UQCRC2 (*p* = 0.4) and ATP5A (*p* = 0.92) (Figure 3). 

Steady-state levels of the mitochondrial outer membrane voltage-dependent anion-selective channel protein 1 (VDAC1) were also reduced in untreated MUT cybrids compared to untreated CON cybrids (58.5%, *p* < 0.01) (Figure 3).

Seven days of PioG + dNs treatment significantly increased NDUFB8 steady-state levels by 81.9% in CON cybrids (*p* < 0.05), whereas levels of SDHA, COII, UQRCR2, and ATP5A were unchanged (Figure 3). In MUT cybrids, PioG + dNs treatment did not alter the steady-state levels of any of the OXPHOS proteins analysed (Figure 3).

Native protein analysis revealed significantly reduced steady-state levels of mature complex I (CI, *p* < 0.001), complex II (CII, *p* < 0.05), the complex III dimer (CIII_2_, *p* < 0.05), the complex III_2_/IV supercomplex (CIII_2_/CIV, *p* < 0.05), complex IV (CIV, *p* < 0.05), and the CI/CIII_2_/CIV supercomplex (*p* < 0.05) in untreated MUT cybrids compared to CON cybrids, as would be expected for this m.3243A>G tRNA mutation [31] (Figure 4). Conversely, increased amounts of the unassembled complex III subunit UQCRC1, detected as a 49.1 kDa monomer following mitochondrial import and processing on BN-PAGE, was increased 2.8-fold in untreated MUT cybrids compared to untreated CON cybrids (*p* < 0.005) (Supplemental Figure S1). There were no differences in the steady-state levels of the translocase of the outer membrane (TOM) complex between CON and MUT cybrid cells, either with or without treatment (Figure 4). The TOM complex is a useful marker of total mitochondrial protein, and demonstrates even loading across the samples, even though differences in OXPHOS complex levels exist (as described below).

PioG + dNs treatment for seven days had a significant effect on OPXHOS complex steady-state levels in CON cybrids, with increases of mature complex I (CI, *p* < 0.05), complex II (CII, *p* < 0.05), the complex III dimer (CIII_2_, *p* < 0.05), the complex III_2_/IV supercomplex (CIII_2_/CIV, *p* < 0.05), complex IV (CIV, *p* < 0.05), complex V (CV, *p* < 0.05), and the CI/CIII_2_/CIV supercomplex (*p* < 0.05) (Figure 4). In contrast, no increase in the steady-state levels of the mature OXPHOS complexes or supercomplexes were observed in MUT cybrids following seven days of PioG + dNs treatment, although levels of complex I and complex V were slightly (but significantly) reduced (Figure 4). 

### 2.4. Treatment with PioG + dNs Increases Mitochondrial Respiratory Capacity in both Control Cybrids and MUT Cybrids

**Figure 5:**
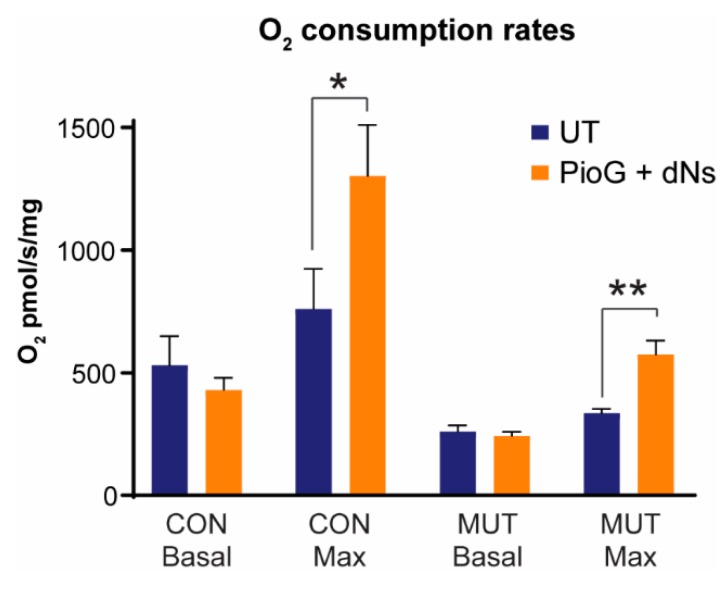
Effect of seven days of PioG + dNs combination treatment on mitochondrial respiration in control (CON) cybrids and m.3243A>G (MUT) cybrids. Basal and maximal respiration rates in untreated MUT cybrids were 49% and 44% of respective untreated CON cybrid rates. PioG + dNs treatment did not affect basal respiration rates in either cell type, but increased maximal respiration rates by 71% in both CON and MUT cybrids. UT = untreated. Values shown are mean ± s.d. * *p* < 0.05, ** *p* < 0.01. Statistical calculations are comparing treated cybrids to their respective untreated controls (Student’s *t*-tests).

We next tested whether treatment with PioG + dNs increases mitochondrial oxygen consumption rates in CON and MUT cybrids. Oxygen flux was measured in intact cells in the presence of glucose (basal respiration rates), glucose and oligomycin (proton leak), or glucose and carbonyl cyanide-4-(trifluoromethoxy)phenylhydrazone (FCCP) (maximal respiration rates). In untreated cells, basal respiration in MUT cybrids was only 49.1% of CON cybrid levels (*p* < 0.05), in agreement with previous reports for this mutation [32] (Table 1 and Figure 5). Similarly, maximal respiration rates were reduced in untreated MUT cybrids to 44.1% of untreated CON cybrids levels (*p* < 0.05). Proton leak rates were also lower in untreated MUT cybrids (68.6% of untreated CON, *p* < 0.05). 

Following seven days of PioG + dNs treatment, basal respiration rates were no different in CON cybrids (*p* = 0.25) or MUT cybrids (*p* = 0.38) compared to their respective untreated controls (Table 1 and Figure 5). However, maximal respiration rates were significantly increased by 71.3% in CON cybrids (*p* < 0.05) and 71.4% in MUT cybrids (*p* < 0.005) following PioG + dNs treatment (Table 1 and Figure 5). Notably, PioG + dNs treatment increased maximal respiration in MUT cybrids from 44.1% to 75.5% of the untreated CON cybrid maximal rate.

PioG + dNs treatment also increased spare respiratory capacity in both CON cybrids (278%, *p* < 0.05) and MUT cybrids (342%, *p* < 0.005), indicating that the bioenergetic limit of these cells is now much higher than their basal rates. Cell respiratory control ratios (a measure of the cell’s capacity for substrate oxidation relative to proton leak; analogous to the mitochondrial respiratory control ratio) were also significantly increased in CON cybrids (55%, *p* < 0.05) and MUT cybrids (71%, *p* < 0.005) (Table 1). 

Overall, these findings reveal that PioG + dNs treatment can increase respiratory chain capacity in both CON and MUT cybrids while maintaining high coupling efficiency for ATP generation. 

## 3. Discussion

The lack of an effective cure for mitochondrial disease has driven the development of new therapies that focus on protecting the mitochondria (and cell) from oxidative damage and the restoration of mitochondrial metabolic activity [5,33]. In this regard, a promising approach is to stimulate mitochondrial biogenesis, boosting the total mitochondrial mass per cell to restore ATP generation above a critical disease threshold. This may be particularly effective for diseases associated with heteroplasmic mtDNA mutations, where residual mitochondrial function persists [34]. Exogenous compounds, such as AICAR, bezafibrate, and resveratrol, can stimulate mitochondrial biogenesis via activation of the PPARγ/PGC-1α pathway, and thus have been considered as potential leads for mitochondrial disease treatment [35]. Unfortunately, clinical trials of these compounds have exhibited limited therapeutic value. AICAR can stimulate mitochondrial proliferation via the AMP-activated protein kinase (AMPK) pathway in vitro [36,37,38], but has shown poor drug potential due to its short half-life in vivo [39]. Bezafibrate, a pan-PPAR agonist, can induce mitochondrial biogenesis and restore mitochondrial function in patient-derived fibroblasts, but was not able to improve symptoms in patients with mitochondrial fatty acid β-oxidation deficiencies [40,41]. Resveratrol, a naturally occurring chemical compound found in the skin of grapes and berries [42], induces mitochondrial biogenesis via Sirtuin 1 (SIRT1) deacetylation of PGC-1α [43,44]. However, high resveratrol doses are associated with negative side effects that may limit its efficacy [45].

In light of these issues, new compounds that stimulate mitochondrial biogenesis are now being trialled for the treatment of various metabolic disorders, including the NRF2 transcription factor activators Epicatechin and RTA408 for Friedrich’s ataxia and mitochondrial myopathy, as well as the turmeric-derived diarylheptanoid curcumin for Leber hereditary optic neuropathy (LHON) [23,35].

Here, we have used a combination of pioglitazone and deoxyribonucleosides (PioG + dNs) to stimulate mitochondrial biogenesis in cybrid cells containing either wild-type (CON) mtDNA or the m.3243A>G MELAS mutation (MUT). Both PioG and dNs have previously been shown to increase mtDNA copy number when used independently [22,29], and we show here for the first time that they can act synergistically to increase mtDNA copy number and mitochondrial mass in both CON and m.3243A>G MUT cell lines. However, the combination treatment did not affect cell proliferation, suggesting the concentrations of PioG and dNs used in this study are non-toxic in vitro.

In untreated m.3243A>G MUT cybrids, the expression of nuclear transcription factors and mtDNA-encoded proteins was increased compared to untreated CON cybrids, most likely as a compensatory response to address the OXPHOS defect caused by the m.3243A>G mutation in these cells. Increased mtDNA transcription has been observed previously in m.3243A>G cybrids [34,46] and m.3243A>G patient-derived induced pluripotent stem (iPS) cells [47]. However, changes to mtDNA transcription, which can either increase or decrease, appears to be dependent on m.3243A>G heteroplasmic levels and cell/tissue type [34,47]. Here, we observed increased mtDNA transcription in cybrids containing >90% m.3243A>G mutation, whereas other reports have observed the highest transcript levels in the presence of either ~33% [46] or ~60% [34] m.3243A>G mutant. 

While mtDNA copy number was increased in both CON and m.3243A>G MUT cybrid cells following PioG + dNs treatment, the subsequent effects on mtDNA transcription were significantly different. In CON cybrids, PioG + dNs treatment increased the expression of nuclear transcription factors associated with mitochondrial biogenesis, namely NRF1, TFAM, and PGC-1α, with a concomitant increase in almost all mtDNA-encoded transcripts examined, including complex I, III, IV, and V subunit-encoding mRNAs. 

In contrast to CON cybrids, combination PioG + dNs treatment of m.3243A>G MUT cybrid cells reduced overall transcription factor and mtDNA transcript expression in MUT cybrids. Interestingly though, transcripts were reduced to similar levels as observed in treated CON cybrids, suggesting that PioG + dNs treatment may be “resetting” expression in MUT cybrids back to an optimal level for respiratory function.

Although mtDNA transcript levels were higher in untreated m.3243A>G MUT cybrid cells compared to untreated CON cybrids, steady-state levels of individual OXPHOS complex I, II, and IV subunits were significantly lower, with no difference in complex III and V subunits. Native PAGE analysis also revealed reduced steady-state levels of mature complex I, complex III, complex IV, and the OXPHOS supercomplex in untreated m.3243A>G MUT cybrids compared to untreated CON cybrids, with similar levels of complex V. Comparable decreases in mature OXPHOS complex levels have been described in m.3243A>G mutant myoblasts, although in these cells complex V was also reduced, again highlighting cell type-dependent phenotypic differences in m.3243A>G mutant expression [48]. 

Interestingly, levels of the complex III subunit UQCRC1, which can be detected as a monomer on native PAGE, were increased in untreated m.3243A>G MUT cybrid cells compared to CON cybrids. This finding suggests that UQCRC1 is stable in monomeric form, even though it has not been incorporated into mature complex III assemblies. As such, caution should be taken when using individual OXPHOS protein subunits as a representation of mature OXPHOS complexes, as the steady-state levels of individual subunits may differ significantly from their respective mature holocomplexes.

Indeed, PioG + dNs treatment did not increase individual OXPHOS subunit levels in CON cybrids (except for the complex I subunit NDUFB8), yet the steady-state levels of mature OXPHOS complexes and supercomplexes were increased. This suggests that PioG + dNs treatment stimulates OXPHOS complex biogenesis by enhancing the assembly of subunits into their respective mature OXPHOS holocomplexes to increase steady-state complex levels, and that this increase is not dependent on higher mtDNA-encoded subunit protein levels per se. The overall result of PioG + dNs treatment in CON cybrids was a 71% increase in maximal mitochondrial respiration, with a 278% increase in spare respiratory capacity and a 55% increase in the cell respiratory control ratio. These results underline how stimulating mitochondrial biogenesis in CON cybrids elevates OXPHOS complex steady-state levels, and how this is associated with a concomitant increase in respiratory flux in these cells. 

PioG + dNs treatment also increased maximal mitochondrial respiration, spare respiratory capacity, and the cell respiratory control ratio in m.3243A>G MUT cybrids; however, steady-state OXPHOS complex levels were not increased as they were in CON cybrids. This suggests that although mitochondrial mass and respiratory capacity are increased in MUT cybrids, PioG + dNs treatment is not stimulating mitochondrial biogenesis via classical pathways in these cells. Instead, the increase in respiratory flux may be due to an altered ubiquinone pool redox status, which has been shown previously to regulate the degradation of complex I [49]. Indeed, others have shown that complex I is cleared by autophagy in differentiated neurons containing high m.3243A>G mutant levels [47], and thus complex I activity may be enhanced by modulating the ubiquinone pool to reduce turnover. Alternatively, PioG + dNs treatment may help to stabilize mtDNA translation, as overexpression of the mitochondrial translation elongation factors EFTu and EFG2 can partially rescue the OXPHOS deficiency in myoblasts containing the m.3243A>G mutation [48]. 

Overall, PioG + dNs treatment appears to increase mtDNA copy number and mitochondrial mass in m.3243A>G MUT cybrids, although steady-state OXPHOS complex levels are not increased. Nevertheless, PioG + dNs treatment is able to successfully increase maximal mitochondrial respiration by 71%, spare respiratory capacity by 342%, and the cell respiratory control ratio by 71% in MUT cells. Importantly, this increases the maximal respiration rate to ~75% of untreated CON cybrid levels, a value which may push respiratory capacity above the critical disease threshold associated with the m.3243A>G mutation [34]. 

In conclusion, we have used a combination of PioG + dNs to increase mitochondrial respiratory capacity in cybrid cells containing >90% of the m.3243A>G mutation associated with MELAS. While mitochondrial biogenesis was not stimulated in m.3243A>G cybrids via classical mechanisms (as we observed in CON cybrids), our findings highlight the ability of PioG + dNs to increase mitochondrial mass and respiratory function in cells harbouring pathogenic mtDNA mutations, as well as the potential for PioG + dNs to be developed into future mitochondrial disease therapies.

## 4. Materials and Methods

### 4.1. Chemicals

Stock solutions were prepared as follows: 20 mM pioglitazone hydrochloride (PioG, Sigma-Aldrich, St. Louis, MO, USA) in DMSO; 50 mM 2′-deoxyguanosine monohydrate (dG, Sigma-Aldrich) in 50% *v*/*v* DMSO; 10 mM 2′-deoxycytidine (dC, Sigma-Aldrich) in distilled H_2_O; 10 mM 2′-deoxyadenosine monohydrate (dA, Sigma-Aldrich) in distilled H_2_O; and 10 mM 2′ deoxythymidine (dT, Sigma-Aldrich) in distilled H_2_O.

### 4.2. Cell Lines and Culture Conditions

The 143BTK^-^ osteosarcoma transmitochondrial cybrids containing either wild-type mitochondrial DNA (mtDNA) or the m.3243A>G *MT-TL1* (tRNA^Leu^) mutation associated with mitochondrial encephalomyopathy, lactic acidosis, and stroke-like episodes (MELAS) were produced by fusion of enucleated lymphoblasts with the 143BTK^-^ ρ^0^ cell line, as described previously [50]. We used lymphoblasts from a control subject and a MELAS patient as mitochondrial donor cells, obtained with written informed consent (St. Vincent’s Hospital, Melbourne, Australia, Human Research Ethics Committee approval #50/97). Both wild-type control (CON) and MELAS patient m.3243A>G mtDNA (MUT) belong to haplogroup T2b (16126C, 16294T, 16296T, 16304C). 

Cybrids were cultured in supplemented Dulbecco’s Modified Eagle Medium (DMEM) media containing 5% (*v*/*v*) fetal calf serum (FCS), 50 units/mL penicillin, 50 μg/mL streptomycin, 50 μg/mL uridine, and 1× GlutaMAX (Thermo Fisher Scientific, Waltham, MA, USA) at 37 °C/5% CO_2_.

Cells were treated with either 10 µM pioglitazone (PioG), a combination of four deoxyribonucleosides (dNs) containing 50 µM dG, 1 µM dC, 1 µM dA, and 1 µM dT, or a combination of both 10 µM PioG and dNs (50 µM dG, 1 µM dC, 1 µM dA, and 1 µM dT) at 37 °C/5% CO_2_ for seven days in supplemented DMEM media. Untreated (UT) controls were incubated with DMSO only at the relevant solute concentrations. Cell counts were performed by haemocytometer.

### 4.3. Mitochondrial DNA (mtDNA) Copy Number Quantitation

MtDNA copy number was determined as described, by calculating the ratio of mtDNA to nuclear DNA (*β-globin* gene) per cell in relation to standard curves of known concentrations, ranging from 10^−1^ ng/µL to 10^−8^ ng/µL [51]. Quantitative PCR was performed on a Rotor Gene RG-3000 (Corbett Research, Mortlake, Australia), with RotorGene software (v6.0) used for data acquisition and analysis. Standard curves were used with a correlation coefficient of *R*^2^ > 0.9 and an efficiency coefficient of *R* > 0.95.

Mitochondrial DNA copy number per cell was calculated as follows:

(1) $$mtDNA\;copies\;(N_{mtDNA})=\frac{\left(ng/µL\right)\times \left(6.023\times 10^{14}\right)}{152\;bp\times 660}$$

(2) $$\beta -globin\;copies\;(N_{\beta -globin})=\frac{\left(ng/µL\right)\times \left(6.023\times 10^{14}\right)}{268\;bp\times 660}$$

(3) $$mtDNA\;copy\;number\;per\;cell=\frac{\;N_{mtDNA}}{0.5\times N_{\beta -globin}}$$

### 4.4. Quantification of m.3243A>G MT-TL1 Mutant Load 

MtDNA PCR amplicons were generated using primers (forward 5′-CCCGATGGTGCACCGC-3′ and reverse 5′-GCATTAGGAATGGCCATTGCG-3′) and cycling conditions of 94 °C for 2 min, 94 °C for 30 s, 55 °C for 30 s, 72 °C for 30 s for 35 cycles, and 72 °C for 10 min. Purified PCR amplicons were sequenced using the forward primer at the Monash Health Translation Precinct (MHTP) Medical Genomics Facility at the Hudson Institute of Medical Research (Melbourne, Australia). Electropherogram data was analysed using QSV Analyser software to quantify mutant peak volumes. 

### 4.5. MtDNA Haplogroup Determination

Mitochondrial haplogroups were determined by PCR and Sanger sequencing of the mitochondrial D-loop hypervariable regions (HVR1, m.15978-16420) and HVR2 (m.8-458) using the following primers: HVR1 forward 5′-CACCATTAGCACCCAAAGCT-3′; HVR1 reverse 5′-TGATTTCACGGAGGATGGTG-3′; HVR2 forward 5′-GGTCTATCACCCTATTAACCAC-3′; and HVR2 reverse 5′-GGGAAAATAATGTGTTAGTTG-3′. Haplogroups were assigned using MitoMaster [52]. 

### 4.6. Assessment of Mitochondrial Mass

Cells were stained with 20 nM tetramethylrhodamine, methyl ester perchlorate (TMRM), 5 µg/mL (*w*/*v*) Fluo-4 AM, 2 µg/mL (*w*/*v*) Hoechst 33342, 10 µM verapamil, and 0.005% Pluronic F-127 in record solution (RS; 109 mM NaCl, 50 mM KCl, 2 mM MgSO_4_, 1.25 mM KH_2_PO_4_, 10 mM d-glucose, 2 mM CaCl_2_, 10 mM HEPES; pH to 7.35) at 37 °C/5% CO_2_ for 1 h, then imaged in 20 nM TMRM and 10 µM verapamil in RS [53]. Forty cells were randomly selected and imaged using a 100× oil objective on a SP5C confocal microscope (Leica, Wetzlar, Germany). The total cytoplasmic volume of each cell was calculated from a single optical layer using ImageJ software by subtracting the nuclear volume (Hoechst 33342 signal) from the total cell volume (Fluo-4 AM cytoplasmic calcium signal). Mitochondrial volume was measured using the TMRM signal and expressed as a percentage of cytoplasmic volume. Background was removed by converting the image to 16-bit greyscale, and a threshold minimum of 40 applied with the dark background setting was selected (to remove cytoplasmic TMRM signal). Cell groups were blinded to eliminate imaging and analysis bias. 

### 4.7. Real-Time Quantitative RT-PCR Analysis

RNA was purified from cybrid cells using an RNeasy Plus Mini Kit (Qiagen, Hilden, Germany) according to manufacturer’s instructions. cDNA was synthesized using the QuantiTect Reverse Transcription Kit (Qiagen), and was used as a template in the subsequent quantitative RT-PCR (qRT-PCR) that was performed using a 7500 Real-Time PCR System (Applied Biosystems, Foster City, CA, USA). qRT-PCR was performed using TaqMan Fast Advanced master mix (Thermo Fisher Scientific) and TaqMan gene expression assays (Thermo Fisher Scientific) for MT-ND1 (Hs02596873), MT-ND2 (Hs02596874), MT-ND3 (Hs02596875), MT-ND4L (Hs02596877), MT-ND4 (Hs02596876), MT-ND5 (Hs02596878), MT-ND6 (Hs02596879), MT-CO1 (Hs02596864), MT-CO3 (Hs02596866), MT-ATP6 (Hs02596862), MT-CYB (Hs02596867), NRF1 (Hs00602161), TFAM (Hs01073348), PGC-1α (Hs00173304), and ACTB (Hs01060665), according to the manufacturer’s instructions. PPARα transcript levels were assessed using a Rotor-gene 3000 (Corbett Research, Mortlake, Australia), as described [19,54]. All reactions were carried out using three independent samples in triplicate. The relative quantitation (relative RNA level) was obtained by applying the comparative Ct method (ΔΔCt), whereby the mRNA expression of each mitochondrial transcript was normalized against the level of ACTB and expressed relative to control. 

### 4.8. Denaturing Gel Electrophoresis

Proteins was separated using denaturing gel electrophoresis, as previously described [55]. In brief, 50 μg of total cell protein was separated on a 12%–18% (*w*/*v*) Tris-tricine continuous gradient gel at 100 V/25 mA for approximately 14 h.

### 4.9. Native Gel Electrophoresis

Blue-native polyacrylamide gel electrophoresis (BN-PAGE) was performed as previously described [55]. In brief, 50 μg of total cell protein was solubilised for 30 min on ice in 50 μL of 20 mM Bis-Tris (pH 7.4), 50 mM NaCl, and 10% (*v*/*v*) glycerol containing either 1% (*v*/*v*) Triton X-100 (Sigma-Aldrich) or 1% (*w*/*v*) digitonin (Merck, Branchburg, NJ, USA). Samples were spun at 16,000× *g* for 5 min, 4 °C to pellet insoluble material, and the supernatant was combined with 5 μL of 10× BN-PAGE loading dye (5% (*w*/*v*) Coomassie Blue G, 500 mM ε-amino-n-caproic acid). Samples were resolved on a 4%–13% (*w*/*v*) BN-PAGE gel at 100 V/5 mA for approximately 14 h at 4 °C.

### 4.10. Western Blotting

Semi-dry Western transfer and immune decoration was performed as previously described [56]. Proteins were visualized with a Chemidoc XRS imaging system (Bio-rad, Hercules, CA, USA). Primary antibodies used were against β-actin (Sigma-Aldrich, A2228), mitochondrial oxidative phosphorylation (OXPHOS) proteins (“Mitoprofile” total OXPHOS Antibody Cocktail, Abcam, Cambridge, UK, ab110411), TOMM40 (Santa Cruz Biotechnology, Dallas, TX, United States, SC-11414), SDHA (Abcam, ab14715), ATP5A (Abcam, ab14748), UQCRC1 (Abcam, ab110252), COI (Abcam, ab14705), and NDUFA9 (raised in rabbits, as previously described [57]). Protein band intensities were calculated using ImageJ software (National Institutes of Health, USA) from three independent, non-saturated images. 

### 4.11. Measurement of Oxygen Consumption Rates

High-resolution respirometry was performed with an Oxygraph-2K oxygen electrode (Oroboros, Innsbruck, Austria). Basal respiration in intact cells was measured in supplemented DMEM, with non-phosphorylating respiration (proton leak) measured in the presence of 5 µg/mL oligomycin and maximal respiration determined by the sequential addition of 1 μM aliquots of carbonyl cyanide-4-(trifluoromethoxy)phenylhydrazone (FCCP). Non-mitochondrial respiration was measured in the presence of 2 μM antimycin A. Spare respiratory capacity (maximal rate – basal rate) and cell respiratory control ratios (maximal/proton leak) were calculated according to [58], using DatLab software (version 4.51, Oroboros Instruments, Innsbruck, Austria) and expressed as pmol O_2_/s/mg of the whole cell protein. 

### 4.12. Statistical Analysis 

All data are expressed as mean ± standard deviation (s.d.), with statistical analysis performed with GraphPad PRISM 7.04. Two-way ANOVA, in combination with Tukey’s post-hoc tests, was used for multiple comparisons, while two-tailed Student’s *t*-tests were used for statistical comparison of two groups. For all analyses, *n* ≥ 3. 

## Figures and Tables

**Table 1:** Respiration rates in untreated and PioG + dNs-treated control and mutant cybrids.

	Basal pmol O_2_/s/mg	Maximal pmol O_2_/s/mg	Proton Leak pmol O_2_/s/mg	Spare Respiratory Capacity	Cell Respiratory Control Ratio
CON UT	530 ± 120	761 ± 163	59 ± 3	231 ± 250	13 ± 3
CON PioG + dNs	431 ± 49	1303 ± 207 *	65 ± 10	872 ± 200 *	20 ± 3 *
MUT UT	260 ± 26 *	335 ± 18 *	41 ± 11 *	75 ± 13	8 ± 0.4 *
MUT PioG + dNs	242 ± 17	575 ± 57 ^###^	41 ± 5	332 ± 45 ^###^	14 ± 1 ^###^

CON: control cybrids; MUT: m.3243A>G mutant cybrids; UT: untreated; PioG + dNs: pioglitazone + deoxyribonucleosides. Spare respiratory capacity equals the maximal rate minus the basal rate; the cell respiratory control ratio equals the maximal rate divided by the proton leak rate. * *p* < 0.05 relative to CON UT values; ^###^ *p* < 0.005 relative to MUT UT values (Student’s *t*-tests).

## Supplementary Materials

The following are available online at https://www.mdpi.com/1422-0067/21/6/2139/s1, Figure S1. Effect of seven days of PioG + dNs combination treatment on native UQCRC1-containing complexes in control (CON) and m.3243A>G (MUT) cybrids.
