# Supplementary material for: Pioglitazone and Deoxyribonucleoside Combination Treatment Increases Mitochondrial Respiratory Capacity in m.3243A>G MELAS Cybrid Cells
Source: Int J Mol Sci. 2020 Mar 20;21(6):2139. doi: 10.3390/ijms21062139 (PMC7139379; doi:10.3390/ijms21062139)
Supplement: Supplementary file 1 [file ijms-21-02139-s001.pdf]

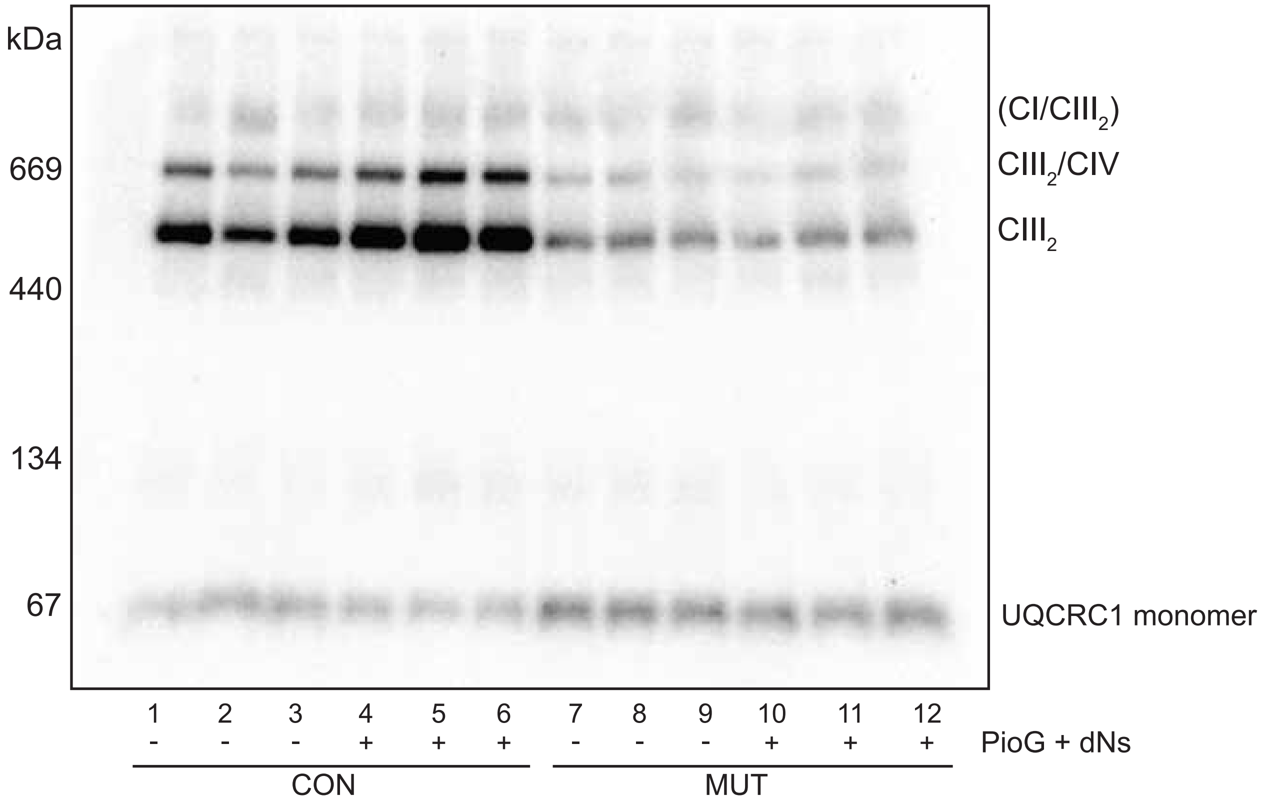

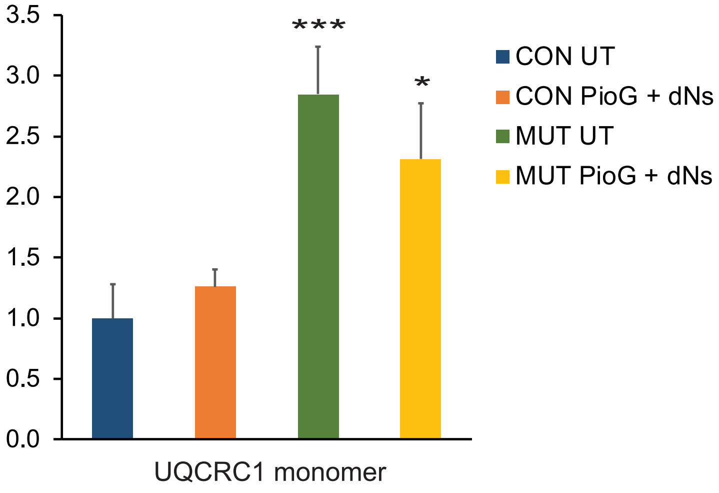

Supplemental Figure S1. Effect of 7 days PioG + dNs combination treatment on native UQCRC1-containing complexes in control (CON) and m.3243A>G (MUT) cybrids. Levels of the complex III dimer (CIII$_2$) and the CIII$_2$/CIV supercomplex were lower in untreated MUT cybrids compared to CON cybrids (as shown in Figure 3). Conversely, levels of UQCRC1 monomer were 2.8x greater in untreated MUT cybrids compared to CON cybrids. PioG + dNs treatment did not alter the levels of UQCRC1 monomer in either CON or MUT cybrids. UT, untreated. Values shown are mean ± s.d. *$p<0.05$, ***$p<0.005$. Statistical calculations are relative to untreated CON values.
